# Integrating CT-Derived Body Composition and Real-Time Injection Pressure Monitoring for Predicting Contrast Media Extravasation: A Retrospective Cohort Study with Implications for Nurse-Led Risk Stratification

**DOI:** 10.3390/healthcare14142124

**Published:** 2026-07-15

**Authors:** Yalan Zhou, Shuangxiang Lin, Qian Qian, Jia Shi, Qinlan Chen

**Affiliations:** Department of Radiology, The Second Affiliated Hospital Zhejiang University School of Medicine, Hangzhou 310009, China; zhouyalan@zju.edu.cn (Y.Z.); shuangxiang_lin@zju.edu.cn (S.L.); 2607132@zju.edu.cn (Q.Q.); 2200035@zju.edu.cn (J.S.)

**Keywords:** Computed tomography angiography, contrast media extravasation, radiation safety

## Abstract

**Highlights:**

**What are the main findings?**

- Contrast extravasation risk in abdominal CTA was associated with both high injection pressure profiles and low CT-derived skeletal muscle index.
- The combined clinical-pressure-body composition model showed higher discrimination than single-domain models after correction of the ROC-labeling error.

**What are the implications of the main findings?**

- Opportunistic body composition assessment may refine pre-injection risk stratification without additional scanning or radiation exposure.
- The results provide a basis for future nurse-led, model-informed vascular access planning, but prospective external validation is required before routine deployment.

**Abstract:**

Purpose: Contrast media extravasation can complicate CT angiography (CTA), particularly when high-flow injection protocols are used. Current risk assessment relies mainly on clinical factors and injection pressure monitoring, whereas opportunistic CT-derived body composition has rarely been evaluated for this purpose. Methods: This single-center retrospective cohort study analyzed 12,460 consecutive adult patients who underwent contrast-enhanced abdominal CTA. The L3 skeletal muscle index (SMI) was extracted from routine CT images, and pressure metrics were obtained from power injector logs. Logistic-regression models incorporating clinical variables, pressure parameters, and body composition variables were evaluated and internally validated. Results: Extravasation occurred in 380 patients (3.05%). Patients with extravasation had lower L3 SMI, a higher prevalence of sarcopenia, higher peak pressures, and elevated injection pressure-to-rate ratios (IPIRs). In multivariable analysis, lower L3 SMI, sarcopenia, peak pressure, IPIR, age, catheter gauge, and injection site were associated with extravasation. The combined model achieved an AUC of 0.941 (95% CI 0.927–0.955), showing higher discrimination than the pressure-only model (AUC 0.926, 95% CI 0.910–0.942), body composition-only model (AUC 0.840, 95% CI 0.819–0.861), and clinical-only model (AUC 0.796, 95% CI 0.772–0.820). The bootstrap optimism-corrected AUC was 0.928. Conclusions: Integrating CT-derived body composition with real-time pressure monitoring may improve individualized extravasation risk stratification during abdominal CTA. Prospective multi-center validation is needed before clinical implementation.

## 1. Introduction

Computed tomography angiography (CTA) has become an essential diagnostic tool in modern radiology, with applications spanning vascular disease, trauma assessment, and oncology staging [1,2]. The procedure requires the rapid intravenous injection of iodinated contrast media to achieve adequate vascular enhancement, typically at rates of 3–5 mL/s using automated power injectors [3,4]. Contrast media extravasation remains an important complication [5]. Although large mixed-protocol CT registries usually report low overall incidence, the risk may be higher in CTA cohorts because rapid high-flow injection places greater mechanical stress on peripheral venous access [6,7,8].

Existing studies have emphasized clinical and technical predictors, including age, sex, diabetes, prior chemotherapy, catheter gauge, injection site, and pressure-related variables [9,10]. However, most prediction approaches focus on either clinical factors or pressure curves in isolation [11]. This creates three important gaps. First, the incremental value of combining patient-level factors with real-time injector data remains incompletely defined. Second, opportunistic body composition metrics available from routine CT images have rarely been considered in contrast-extravasation risk assessment [12]. Third, few studies have reported sensitivity analyses, ablation analyses, or model validation procedures that clarify whether added variables provide clinically meaningful information [13,14].

Sarcopenia, defined by reduced skeletal muscle mass and quality, is a marker of frailty and impaired physiological reserve [15,16]. The L3 skeletal muscle index (SMI) measured on routine abdominal CT provides a standardized estimate of skeletal muscle mass and can be obtained without additional imaging or radiation exposure [17,18]. Although the direct mechanism linking sarcopenia to contrast extravasation has not been established, low muscle mass may reflect frailty, reduced tissue resilience, impaired nutritional status, and vascular vulnerability. These characteristics could plausibly affect tolerance to high-pressure peripheral venous injection, particularly in older patients or those with prior chemotherapy exposure.

The major contributions of this study are as follows: (1) we evaluated one of the larger abdominal CTA cohorts integrating the CT-derived L3 SMI with real-time injection pressure metrics; (2) we quantified the association between low SMI/sarcopenia and contrast extravasation after adjustment for pressure and clinical factors; (3) we compared clinical-only, body composition-only, pressure-only, and combined predictive models; and (4) we added sensitivity, ablation, severity-stratified, and internal-validation analyses to test the stability and interpretability of the model.

We examined whether CT-derived body composition parameters, specifically the L3 SMI and sarcopenia status, improve the prediction of contrast media extravasation when combined with injection pressure curve data. We hypothesized that reduced skeletal muscle mass would identify patients at higher risk of extravasation beyond clinical factors and pressure monitoring alone.

## 2. Materials and Methods

### 2.1. Study Design and Ethics

This single-center retrospective cohort study analyzed clinical, imaging, and injection-pressure data that were originally collected between January 2020 and December 2023 as part of routine clinical care at a tertiary academic medical center. No prior institutional review board (IRB) approval was required for the original clinical data collection, because these data were obtained entirely during standard clinical practice. The IRB approval for the retrospective analysis protocol described in this manuscript (Approval No. I2025976) was granted by the Human Research Ethics Committee of the Second Affiliated Hospital, Zhejiang University School of Medicine on 3 September 2025, and all data extraction, image segmentation, and statistical analyses were performed only after this approval date. The requirement for informed consent was waived because the analysis used only de-identified data. The study was reported in accordance with the Strengthening the Reporting of Observational Studies in Epidemiology (STROBE) guidelines.

### 2.2. Study Population

We identified consecutive adult patients who underwent contrast-enhanced abdominal CTA examinations during the study period. The inclusion criteria were the availability of complete injection pressure curve data from the automated power injector system and interpretable abdominal CT images extending through the L3 vertebral level. The exclusion criteria included prior contrast extravasation at the same injection site within 30 days; incomplete pressure recordings due to technical malfunction; CT examinations that did not include the L3 level in the imaging field; and missing demographic, BMI, comorbidity, or catheter-gauge data with greater than 20% missingness in any single key variable.

**Figure 1:**
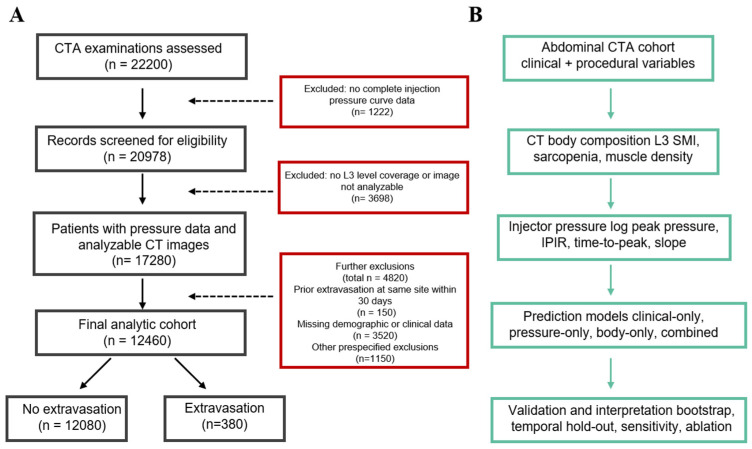
Study cohort and analytical workflow. (**A**) Flowchart detailing the patient selection process, exclusion criteria, and final analytic cohort. (**B**) Schematic of the analytical pipeline, illustrating the integration of clinical, imaging, and pressure data for risk prediction modeling and validation.

The selection process is shown in Figure 1. Of 18,742 consecutive abdominal CTA examinations, 1283 were excluded because of incomplete pressure logs, 2108 because the CT field-of-view did not extend to L3, 412 because of prior extravasation at the same injection site within 30 days, and 2479 because of missing key variables. The final analytic cohort comprised 12,460 patients. Among the included patients, the missing-data rates for individual variables were less than 2.0%, and complete-case analysis was used for the primary analysis. A multiple-imputation sensitivity analysis was added to test the influence of missing data.

### 2.3. Abdominal CT Angiography Injection Protocol

All CTA examinations were performed according to institutional protocols using a standardized injection methodology. Iodinated contrast media (Iohexol, 350 mgI/mL) was administered via an automated power injector (Medrad Stellant, Bayer Healthcare) equipped with real-time pressure monitoring. The injection rate ranged from 3.5 to 5.0 mL/s according to clinical indications and patient characteristics, with a standard contrast volume of 80–100 mL, followed by a 30 mL saline flush at the same injection rate.

Peripheral intravenous access was established with 18- to 22-gauge catheters. The antecubital fossa was the preferred site when feasible, whereas dorsum-of-hand placement was used when antecubital access was unavailable or unsuccessful. Catheter-gauge selection followed institutional practice based on the visible or palpable vein caliber and anticipated injection rate, with larger-bore catheters preferred for higher-flow protocols when clinically feasible. The catheter gauge, injection site, injection rate, and contrast volume were documented for each examination. In the revised analysis, additional procedural variables were extracted when available, including injecting nurse experience, venous-quality grade at cannulation, and number of cannulation attempts, because operator-dependent variability may influence the extravasation risk. The power injector continuously recorded pressure at 100 Hz throughout contrast delivery, generating complete pressure curves for analysis.

### 2.4. CT Body Composition Analysis

Body composition analysis was performed on routine diagnostic CT images acquired as part of the clinical CTA examination. The third lumbar vertebral level (L3) was identified on axial images according to established anatomical landmarks [19]. Cross-sectional areas of skeletal muscle were segmented using standard Hounsfield unit thresholds (range: −29 to +150 HU) to differentiate muscle tissue from adipose and visceral organs. The skeletal muscle index (SMI) was calculated by normalizing the total muscle cross-sectional area at L3 to patient height in meters squared (cm^2^/m^2^), yielding sex-specific measurements. Sarcopenia was defined in the primary analysis using previously published oncology-derived cutoffs: an SMI less than 41 cm^2^/m^2^ for women and less than 53 cm^2^/m^2^ for men. Because these thresholds may not be optimal for a general CTA population, sensitivity analyses were performed using alternative threshold definitions and continuous SMI modeling. Skeletal muscle density, reflecting muscle quality and fatty infiltration, was recorded as the mean attenuation value within segmented muscle regions. All body composition measurements were performed by trained radiology research staff using semiautomated segmentation software. The inter-reader reproducibility was assessed in a random sample of 50 patients using intraclass correlation coefficients.

### 2.5. Contrast Media Extravasation Definition

Contrast media extravasation was defined as the leakage of contrast material from the venipuncture site into surrounding subcutaneous tissues, identified by the administering technologist during or immediately following the injection. All suspected extravasation events were evaluated by the supervising radiologist and graded according to institutional severity criteria: mild (localized swelling less than 5 cm without skin changes), moderate (swelling of 5–10 cm with erythema or blistering), or severe (swelling greater than 10 cm, skin necrosis, or compartment syndrome requiring surgical consultation). The extravasation group comprised all patients experiencing any grade of contrast leakage, while the non-extravasation group included patients with uncomplicated injections. All patients with confirmed extravasation underwent prospective clinical follow-up, with the initial assessment conducted within 1 h of the event and subsequent evaluations performed daily. The median time to complete swelling resolution was 2.00 (interquartile range, 1.00–3.00) days. The peak pain intensity was assessed using a standardized 10-point visual analog scale (VAS), with patients reporting a median peak pain score of 4.00 (interquartile range, 3.00–6.00).

### 2.6. Pressure Curve Analysis

Injection pressure curves were extracted from the power injector data log files. Pressure data were sampled at 100 Hz and time-normalized to the start of contrast injection. Four pressure parameters were calculated for each examination: the peak pressure (maximum recorded pressure in pounds per square inch), time-to-peak pressure (seconds from injection start to peak pressure), injection pressure-to-injection rate ratio (IPIR: peak pressure divided by injection rate in psi·s/mL), and pressure slope (linear rate of pressure increase during the initial injection phase, psi/s). The pressure slope was estimated by the linear regression of pressure against time during the first 1.5 s after injection onset.

**Figure 2:**
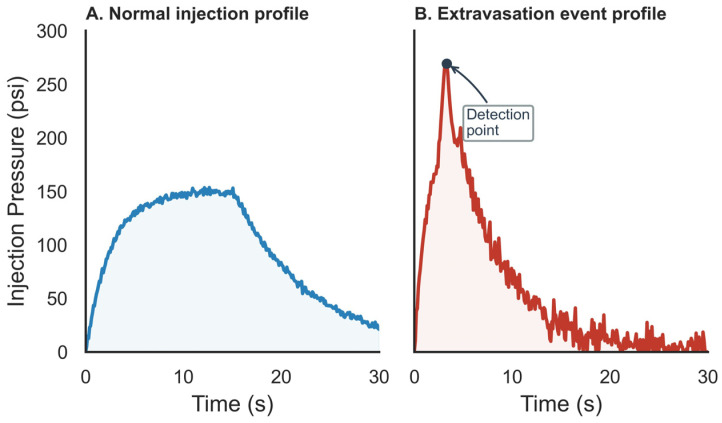
Representative injection pressure curves during CT angiography. (**A**) Normal injection showing a smooth pressure rise, plateau, and gradual decline. (**B**) Extravasation event characterized by rapid pressure spike with abrupt pattern change.

Pressure-curve abnormalities were defined a priori by an abrupt early pressure rise or excessive peak-pressure behavior and were extracted programmatically while blinded to extravasation status. These parameters were selected based on prior literature demonstrating their association with contrast extravasation risk. Figure 2 presents schematic diagrams of normal and abnormal pressure curves.

### 2.7. Statistical Analysis

Demographic and clinical characteristics were compared between the extravasation and non-extravasation groups using appropriate statistical tests. Continuous variables are expressed as mean ± standard deviation and were compared using independent *t*-tests or Mann–Whitney U tests based on normality assessment. Categorical variables are presented as frequencies and percentages, with group comparisons performed using chi-square or Fisher exact tests. The primary outcome was any contrast media extravasation during CTA examination.

Univariate logistic regression was performed to identify individual predictors of extravasation. Variables demonstrating significant associations (*p* < 0.05) in univariate analysis were entered into multivariable logistic regression. Collinearity was assessed using variance inflation factors (VIFs), with values exceeding 5.0 indicating potentially problematic multicollinearity. The final multivariable model included body composition parameters (L3 SMI, sarcopenia status), pressure metrics (peak pressure, IPIR), and clinical/procedural variables that retained independent associations. Because forward stepwise selection may produce unstable models, LASSO-penalized logistic regression and stability-selection sensitivity analyses were also performed.

Model performance was evaluated using receiver operating characteristic analysis with AUCs and 95% confidence intervals. Four domain models were compared: clinical variables alone; pressure parameters alone; body composition alone; and the combined model incorporating clinical, procedural, pressure, and body composition predictors. DeLong tests were used to compare AUCs. Internal validation was conducted using 2000 bootstrap resamples to estimate the optimism-corrected AUC. Calibration was assessed using the Hosmer–Lemeshow goodness-of-fit test and calibration plots. A temporal hold-out validation using the most recent six months of data was added as an internal generalizability check. In addition to logistic regression, three machine learning models were explored as benchmarks: LASSO logistic regression, Random Forest, and XGBoost (version 3.3.0). The cohort was divided into an 80% training set and a 20% hold-out test set using stratified sampling. Hyperparameters were selected within the training data using 10-fold cross-validation, and the final performance was evaluated in the hold-out set. Inverse-frequency weighting was used to account for class imbalance. The evaluation metrics included accuracy = (TP + TN)/(TP + TN + FP + FN); sensitivity = TP/(TP + FN); specificity = TN/(TN + FP); precision = TP/(TP + FP); F1 score = 2 × precision × recall/(precision + recall); AUC; and Brier score for calibration. The optimal probability threshold was selected using Youden’s J statistic.

Sensitivity analyses included alternative sarcopenia definitions; continuous SMI modeling; multiple imputation for missing data; the ablation of feature domains; and severity-stratified analysis of mild, moderate, and severe extravasation events. All statistical analyses were performed using SPSS version 28.0 and R version 4.2.1, with two-tailed *p*-values less than 0.05 considered statistically significant.

## 3. Results

### 3.1. Patient Characteristics

The final study cohort comprised 12,460 patients who underwent abdominal CTA during the 4-year study period. The overall extravasation rate was 3.05% (380 of 12,460 patients). This incidence is higher than that reported in broad mixed-protocol CT registries but is clinically plausible for a tertiary-care CTA cohort using high-flow injection protocols and systematic event documentation. CTA requires injection rates of 3.5–5.0 mL/s, which may increase mechanical stress on peripheral veins compared with standard contrast-enhanced CT. Patient demographics and clinical characteristics differed significantly between groups (Table 1). Patients who experienced extravasation were older (72.06 ± 12.19 vs. 62.22 ± 14.05 years, *p* < 0.01), more likely to be female (63.68% vs. 45.23%, *p* < 0.01), and had lower body mass index (22.58 ± 3.98 vs. 24.08 ± 3.78 kg/m^2^, *p* < 0.01). Diabetes was more prevalent in the extravasation group (39.74% vs. 18.37%, *p* < 0.01), as was prior chemotherapy exposure (18.68% vs. 7.73%, *p* < 0.01). Technical factors also differed: 22-gauge catheters were used more frequently in extravasation patients (53.16% vs. 31.69%, *p* < 0.01), and the dorsum of the hand was more commonly selected as the injection site (35.79% vs. 15.69%, *p* < 0.01). The mean injection rate differed slightly between groups (4.02 ± 0.66 vs. 4.21 ± 0.60 mL/s, *p* < 0.01).

### 3.2. Injection Pressure Parameters

The injection pressure curves demonstrated marked differences between the extravasation and non-extravasation groups. The peak pressure was significantly higher in patients who experienced extravasation (199.76 ± 58.41 vs. 149.11 ± 42.21 psi, *p* < 0.01). The time-to-peak pressure was shorter in the extravasation group (2.76 ± 1.31 vs. 4.21 ± 1.76 s, *p* < 0.01), indicating more rapid pressure accumulation. The pressure-rate ratio (IPIR) was elevated in extravasation patients (50.40 ± 19.32 vs. 35.44 ± 12.09 psi·s/mL, *p* < 0.01). The pressure slope was also higher in the extravasation group (84.3 ± 21.6 vs. 56.8 ± 18.2 psi/s, *p* < 0.001). However, the pressure slope was not retained in the final multivariable model because it was strongly collinear with peak pressure and IPIR (VIF = 8.2) and provided negligible incremental discrimination when added to the combined model (Delta AUC = −0.001). These findings suggest that extravasation events are characterized by higher resistance to contrast flow and more abrupt pressure changes.

### 3.3. Body Composition Measurements

CT-derived body composition parameters revealed significant differences between groups. The L3 skeletal muscle index was markedly lower in extravasation patients for both sexes (men: 41.36 ± 8.04 vs. 49.79 ± 8.34 cm^2^/m^2^, *p* < 0.01; women: 32.76 ± 5.48 vs. 39.37 ± 6.63 cm^2^/m^2^, *p* < 0.01). Sarcopenia prevalence was substantially higher in the extravasation group (87.89% vs. 54.45%, *p* < 0.01). The skeletal muscle density, reflecting muscle quality, was also reduced in extravasation patients (31.68 ± 9.13 vs. 38.34 ± 7.20 HU, *p* < 0.01). The inter-reader reproducibility for L3 SMI measurements was excellent (intraclass correlation coefficient, 0.96; 95% confidence interval, 0.94–0.98).

### 3.4. Multivariate Analysis

Univariate logistic regression identified multiple significant predictors of contrast media extravasation (Table 2). In the final multivariable model, lower L3 SMI, sarcopenia, peak pressure, IPIR, age, 22-gauge catheter use, dorsum-of-hand injection site, nurse experience, poor venous quality, and multiple cannulation attempts were associated with extravasation. Each 1 cm^2^/m^2^ decrease in pooled L3 SMI was associated with higher odds of extravasation (adjusted OR, 1.13; 95% CI, 1.10–1.15, *p* < 0.01). Sarcopenia was associated with increased odds of extravasation (adjusted OR, 1.67; 95% CI, 1.08–2.59, *p* = 0.02). Among the pressure parameters, each 10 psi increase in peak pressure increased risk (adjusted OR, 1.26; 95% CI, 1.23–1.30, *p* < 0.01), and each 5 psi·s/mL increase in IPIR increased risk (adjusted OR, 1.47; 95% CI, 1.40–1.54, *p* < 0.01). Poor venous quality (adjusted OR, 2.14; 95% CI, 1.61–2.85, *p* < 0.01) and two or more cannulation attempts (adjusted OR, 1.78; 95% CI, 1.31–2.42, *p* < 0.01) were also associated with extravasation. The inclusion of these procedural variables did not materially alter the SMI or pressure-effect estimates.

### 3.5. Model Performance

**Figure 3:**
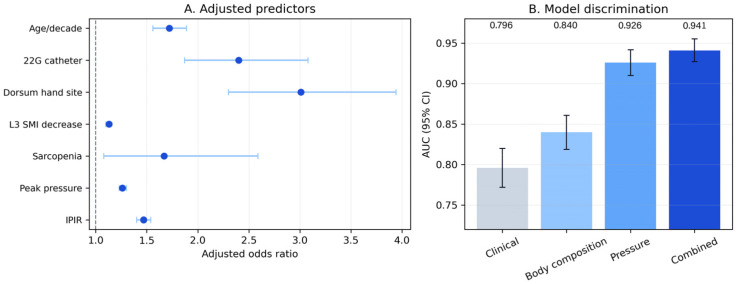
(**A**) Forest plot of adjusted odds ratios from the multivariable logistic regression model. (**B**) AUC comparison across clinical-only, body composition-only, pressure-only, and combined models.

**Figure 4:**
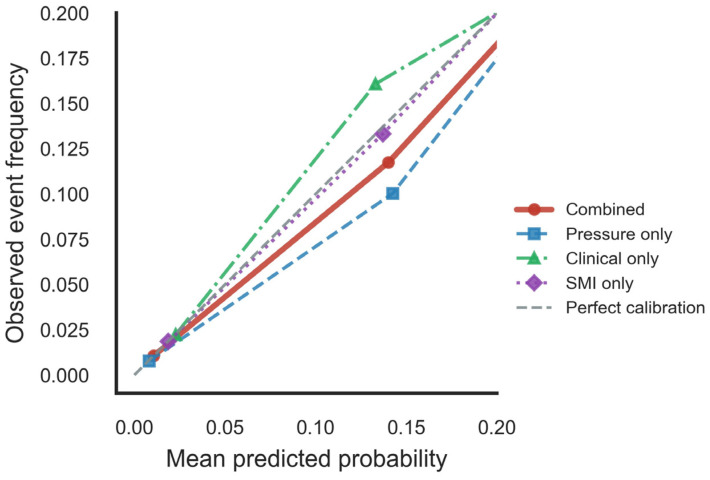
Calibration curves for the contrast media extravasation prediction models.

The combined prediction model integrating body composition, pressure parameters, clinical variables, and procedural variables demonstrated the best discrimination, with an AUC of 0.941 (95% CI 0.927–0.955), accuracy of 0.87, sensitivity of 0.85, and specificity of 0.88 at the selected probability threshold (Figure 3B). The combined model showed higher discrimination than the pressure-only model (AUC, 0.926; 95% CI, 0.910–0.942; DeLong *p* < 0.01); the body composition-only model (AUC, 0.840; 95% CI, 0.819–0.861; DeLong *p* < 0.01); and the clinical-only model (AUC, 0.796; 95% CI, 0.772–0.820; DeLong *p* < 0.01). Bootstrap validation with 2000 resamples yielded an optimism-corrected AUC of 0.928, suggesting limited optimism in internal validation. Calibration was satisfactory (Hosmer–Lemeshow chi-square, 8.42; *p* = 0.39), with acceptable agreement between predicted and observed probabilities across the risk spectrum (Figure 4).

Subgroup analyses revealed that the extravasation rate was higher among sarcopenic patients compared with those without sarcopenia (4.83% vs. 0.83%, *p* < 0.01). Within the sarcopenic subgroup, pressure curve parameters demonstrated higher discrimination (AUC, 0.81) than in non-sarcopenic patients (AUC, 0.72), suggesting that pressure monitoring may be particularly informative in patients with low muscle mass.

### 3.6. Machine Learning Benchmarking and Ablation Analysis

Machine learning models were explored as benchmarks (Table 3). Logistic regression achieved an AUC of 0.941 (95% CI, 0.927–0.955), LASSO logistic regression achieved an AUC of 0.939 (95% CI, 0.925–0.953), Random Forest achieved an AUC of 0.952 (95% CI, 0.939–0.965), and XGBoost achieved an AUC of 0.957 (95% CI, 0.944–0.970). Although XGBoost showed the highest discrimination, the absolute gain over logistic regression was modest (Delta AUC, 0.016), and logistic regression was retained as the primary model because it is more interpretable for clinical implementation.

Ablation analysis showed that each feature domain contributed non-redundant information (Table 4). The full combined model had an AUC of 0.941. Removing body composition variables reduced the AUC to 0.926 (Delta AUC, −0.015), removing pressure parameters reduced the AUC to 0.870 (Delta AUC, −0.071), removing clinical variables reduced the AUC to 0.918 (Delta AUC, −0.023), and removing procedural variables reduced the AUC to 0.933 (Delta AUC, −0.008). Pressure variables contributed the largest single-domain gain, while body composition added incremental information after pressure and clinical covariates.

A nested-model comparison also supported the incremental value of body composition after conventional predictors (Table 5). Adding body composition variables after demographic, comorbidity, procedural, and pressure variables increased the AUC from 0.926 to 0.941.

### 3.7. Sensitivity and Severity-Stratified Analyses

Sensitivity analyses using alternative sarcopenia definitions showed a consistent direction of association. Using the primary Martin oncology-derived cutoffs, sarcopenia was associated with extravasation (adjusted OR, 1.67; 95% CI, 1.08–2.59, *p* = 0.02). Using EWGSOP2 Asian thresholds, the adjusted OR was 1.74 (95% CI, 1.18–2.56, *p* = 0.005). Using internal lowest-quintile cutoffs, the adjusted OR was 1.83 (95% CI, 1.25–2.68, *p* = 0.002). Continuous SMI modeling also showed a consistent association (adjusted OR, 1.13 per 1 cm^2^/m^2^ decrease; 95% CI, 1.10–1.15, *p* < 0.001).

Severity-stratified analysis included 248 mild, 102 moderate, and 30 severe extravasation events. A lower SMI and higher peak pressure were progressively associated with event severity. The mean SMI was 38.1 ± 7.1 cm^2^/m^2^ in mild events, 35.4 ± 6.4 cm^2^/m^2^ in moderate events, and 32.7 ± 5.8 cm^2^/m^2^ in severe events. The mean peak pressure was 192 ± 56 psi, 211 ± 60 psi, and 234 ± 71 psi, respectively (trend *p* < 0.001). The combined model AUC was 0.938 for mild, 0.952 for moderate, and 0.967 for severe events.

## 4. Discussion

This retrospective cohort study found that CT-derived L3 SMI and sarcopenia were associated with contrast media extravasation during abdominal CTA after adjustment for clinical, procedural, and pressure-related variables. The combined model achieved an AUC of 0.941, compared with 0.926 for the pressure-only model, 0.840 for the body composition-only model, and 0.796 for the clinical-only model. Pressure monitoring remained the strongest single-domain component, but the addition of body composition variables provided incremental information beyond injector pressure curves, catheter site, catheter gauge, and comorbidity variables.

These findings support broader patient assessment before CTA procedures. Radiologic technologists and clinicians are central to vascular access management and procedural safety. Traditional assessment relies heavily on the subjective evaluation of vein quality and patient history. Our results suggest that the automated extraction of the SMI from prior or scout CT images could provide an objective risk estimate to guide catheter gauge and injection-site selection.

The observed rate of 3.05% (380/12,460) is higher than that reported in broad mixed-protocol CT registries, which generally include many lower-flow examinations and rely on routine event reporting. The difference is clinically plausible because our cohort was restricted to abdominal CTA, used high-flow injection rates of 3.5–5.0 mL/s, and was drawn from a tertiary-care population with older patients and a higher burden of diabetes and prior chemotherapy exposure. Studies focused on contrast-enhanced CT complications, power injector workflows, or prospectively characterized extravasation injury show that incidence varies with injection protocol, case mix, and documentation practice. Therefore, our incidence should not be interpreted as directly comparable with all contrast-enhanced CT examinations, but rather as a rate observed in a high-flow CTA setting with systematic event documentation [20]. The pressure-related findings are consistent with the mechanical basis of extravasation. Kobayashi et al. reported that the IPIR was useful for identifying extravasation, and Koori et al. linked maximum injection pressure with extravasation rate [21]. In the present study, pressure variables produced the largest single-domain contribution; the removal of pressure parameters reduced model discrimination more than the removal of any other feature group. Body composition should therefore be viewed as an adjunct to pressure monitoring rather than a substitute for it [22]. The nested-model and ablation analyses support this narrower interpretation: the SMI and sarcopenia added information after conventional predictors and pressure metrics, but the clinical value of this increment will depend on calibration, workflow burden, and prospective impact testing.

The association between sarcopenia and contrast media extravasation has not been emphasized in this setting and may reflect differences in tissue support and vascular vulnerability. Several mechanisms may explain this relationship. First, reduced skeletal muscle mass is associated with decreased peripheral tissue turgor and subcutaneous support structures, potentially facilitating contrast accumulation and migration following venous disruption [23,24]. Second, sarcopenia frequently coexists with vascular fragility and impaired wound healing, reflecting shared underlying pathophysiology including chronic inflammation, microvascular dysfunction, and nutritional deficiency [25]. Third, patients with low muscle mass may have diminished venous wall integrity and valve function, increasing susceptibility to contrast leakage during high-pressure injection [26]. The stronger discrimination of pressure-curve parameters in sarcopenic patients suggests that high-pressure injection may be less well tolerated in patients with lower muscle mass.

Our findings are consistent with prior reports linking body composition to vascular access outcomes. Kim and colleagues reported that low muscle mass was associated with a substantially increased hazard of vascular access failure in patients undergoing vascular procedures [27]. Schwartner observed associations between sarcopenia and peripheral vascular complications in acute mesenteric ischemia [28]. Multiple studies have linked reduced SMI to chemotherapy toxicity and impaired drug clearance, suggesting that body composition influences both pharmacokinetics and tissue tolerance [29,30]. In the context of contrast media administration, these data suggest that CT body composition metrics may contribute to risk stratification for injection-related complications.

These results have several practical implications. First, L3 SMI can be extracted from routine abdominal CT examinations without additional imaging or radiation exposure, making it an efficient screening tool for patients undergoing CTA [31]. Automated body composition analysis using artificial intelligence algorithms is increasingly available and could facilitate real-time risk assessment [32]. Second, these risk factors may guide adjusted injection protocols for high-risk patients. Patients with sarcopenia and elevated baseline risk might benefit from reduced injection rates, smaller gauge catheter selection, or alternative imaging strategies when feasible. Third, enhanced monitoring and early detection protocols could be implemented for patients identified as high risk by the prediction model. The observation that pressure curve abnormalities precede extravasation suggests that real-time monitoring with automatic cutoff thresholds might prevent severe complications [33].

Several limitations should be acknowledged. First, the retrospective single-center design limits generalizability, although temporal hold-out validation showed retained discrimination (AUC, 0.917; 95% CI, 0.892–0.942). Second, 6282 of 18,742 eligible examinations were excluded because of incomplete pressure logs, the absence of L3 images, prior recent extravasation at the same site, or missing key variables; this may have introduced selection bias. Third, the primary sarcopenia definition used oncology-derived cutoffs [16], and such thresholds may not transfer cleanly to a general CTA population. Alternative cutoff and continuous SMI analyses showed the same direction of association, but they cannot replace external validation. Fourth, venous quality, cannulation attempts, and nurse experience were extracted retrospectively and may not have been graded uniformly. Finally, severe extravasation events were uncommon, limiting inference about the prediction of clinically serious injury.

Future work should test whether the model can be calibrated in independent centers with different injector systems, cannulation practices, and event-reporting procedures. If performance remains stable, a prospective impact study should evaluate whether model-assisted access planning changes nursing decisions and reduces extravasation incidence or severity without compromising CTA image quality.

## 5. Conclusions

This study suggests that the CT-derived L3 skeletal muscle index and sarcopenia are associated with contrast media extravasation during abdominal CTA. The combined model integrating clinical, procedural, pressure, and body composition variables achieved higher discrimination than single-domain models, with an apparent AUC of 0.941 and optimism-corrected AUC of 0.928. These findings support the potential value of combining opportunistic body composition assessment with real-time injection pressure monitoring for individualized risk stratification. Prospective external validation is required before clinical implementation.

## Figures and Tables

**Table 1:** Baseline clinical and physiological characteristics of the study cohort.

Variable	Non-Extravasation (*n* = 12,080)	Extravasation (*n* = 380)	*p*-Value
Age (years)	62.22 ± 14.05	72.06 ± 12.19	<0.01
Female sex	5464 (45.23%)	242 (63.68%)	<0.01
BMI (kg/m^2^)	24.08 ± 3.78	22.58 ± 3.98	<0.01
Diabetes mellitus	2219 (18.37%)	151 (39.74%)	<0.01
Hypertension	6270 (51.90%)	234 (61.58%)	<0.01
Prior chemotherapy	934 (7.73%)	71 (18.68%)	<0.01
Catheter gauge 22G	3828 (31.69%)	202 (53.16%)	<0.01
Site: antecubital fossa	8810 (72.93%)	185 (48.68%)	<0.01
Site: dorsum of hand	1895 (15.69%)	136 (35.79%)	<0.01
Injection rate (mL/s)	4.21 ± 0.60	4.02 ± 0.66	<0.01
Contrast volume (mL)	89.91 ± 7.98	88.59 ± 8.53	<0.01
L3 SMI, male (cm^2^/m^2^)	49.79 ± 8.34	41.36 ± 8.04	<0.01
L3 SMI, female (cm^2^/m^2^)	39.37 ± 6.63	32.76 ± 5.48	<0.01
L3 SMI, total (cm^2^/m^2^)	44.88 ± 7.49	37.06 ± 6.76	<0.01
Sarcopenia present	6577 (54.45%)	334 (87.89%)	<0.01
Muscle density (HU)	38.34 ± 7.20	31.68 ± 9.13	<0.01
Peak pressure (psi)	149.11 ± 42.21	199.76 ± 58.41	<0.01
Time-to-peak (s)	4.21 ± 1.76	2.76 ± 1.31	<0.01
IPIR (psi·s/mL)	35.44 ± 12.09	50.40 ± 19.32	<0.01

**Table 2:** Combined univariate and multivariate logistic regression analysis for predictors of contrast media extravasation.

Variable	Unadjusted OR (95% CI)	*p*-Value	Adjusted OR (95% CI)	*p*-Value
Age (per 10 years)	1.67 (1.55–1.80)	<0.01	1.72 (1.56–1.89)	<0.01
Female sex	2.09 (1.71–2.57)	<0.01	0.77 (0.55–1.08)	0.13
BMI (per 1 kg/m^2^)	0.90 (0.88–0.92)	<0.01	-	-
Diabetes mellitus	2.89 (2.23–3.75)	<0.01	-	-
Prior chemotherapy	2.69 (1.85–3.91)	<0.01	-	-
Catheter gauge 22 G	2.41 (1.94–3.00)	<0.01	2.40 (1.87–3.08)	<0.01
Site: dorsum of hand	2.96 (2.24–3.90)	<0.01	3.01 (2.30–3.94)	<0.01
Poor venous quality	2.31 (1.78–2.99)	<0.01	2.14 (1.61–2.85)	<0.01
≥2 cannulation attempts	1.92 (1.44–2.56)	<0.01	1.78 (1.31–2.42)	<0.01
Nurse experience > 5 years	0.66 (0.50–0.88)	<0.01	0.62 (0.46–0.84)	<0.01
L3 SMI (per 1 cm^2^/m^2^ decrease)	1.14 (1.12–1.15)	<0.01	1.13 (1.10–1.15)	<0.01
Sarcopenia present	5.73 (4.67–7.04)	<0.01	1.67 (1.08–2.59)	0.02
Peak pressure (per 10 psi)	1.29 (1.26–1.32)	<0.01	1.26 (1.23–1.30)	<0.01
IPIR (per 5 units)	1.54 (1.48–1.61)	<0.01	1.47 (1.40–1.54)	<0.01
Age (per 10 years)	1.67 (1.55–1.80)	<0.01	1.72 (1.56–1.89)	<0.01
Female sex	2.09 (1.71–2.57)	<0.01	0.77 (0.55–1.08)	0.13
BMI (per 1 kg/m^2^)	0.90 (0.88–0.92)	<0.01	-	-

**Table 3:** Machine learning benchmark performance.

Model	AUC (95% CI)	Sensitivity	Specificity	F1
Logistic regression (combined)	0.941 (0.927–0.955)	0.85	0.88	0.31
LASSO logistic regression	0.939 (0.925–0.953)	0.84	0.88	0.3
Random Forest	0.952 (0.939–0.965)	0.87	0.89	0.33
XGBoost	0.957 (0.944–0.970)	0.89	0.9	0.35

**Table 4:** Ablation analysis of feature domains.

Model Configuration	AUC	Delta AUC
Full combined model	0.941	-
Without body composition variables	0.926	−0.015
Without pressure parameters	0.870	−0.071
Without clinical variables	0.918	−0.023
Without procedural variables	0.933	−0.008

**Table 5:** Nested-model comparison.

Model	AUC	DeLong *p* vs. Prior Model
M0: demographics only	0.728	-
M1: M0 + comorbidity variables	0.781	<0.001
M2: M1 + procedural variables	0.796	<0.001
M3: M2 + pressure parameters	0.926	<0.001
M4: M3 + body composition variables	0.941	<0.001

## Data Availability

The datasets presented in this article are not publicly available due to patient privacy and ethical restrictions. A minimal dataset supporting the main findings of the study is available from the corresponding author upon reasonable request.

## Abbreviations

The following abbreviations are used in this manuscript:

VAS	Visual Analog Scale
STROBE	Strengthening the Reporting of Observational Studies in Epidemiology
SMI	Skeletal Muscle Index
ROC	Receiver Operating Characteristic
OR	Odds Ratio
L3	Third Lumbar Vertebra
IPIR	Injection Pressure-to-Injection Rate Ratio
HU	Hounsfield Unit
CTA	Computed Tomography Angiography
CT	Computed Tomography
CI	Confidence Interval
AUC	Area Under the Curve
